# Chatbot for the Return of Positive Genetic Screening Results for Hereditary Cancer Syndromes: Prompt Engineering Project

**DOI:** 10.2196/65848

**Published:** 2025-06-10

**Authors:** Emma Coen, Guilherme Del Fiol, Kimberly A Kaphingst, Emerson Borsato, Jackilen Shannon, Hadley Smith, Aaron Masino, Caitlin G Allen

**Affiliations:** 1 School of Computing, Clemson University, 105 Sikes Hall, Clemson, SC, 29634, United States; 2 Department of Biomedical Informatics, University of Utah, Salt Lake City, UT, United States; 3 Department of Communication, University of Utah, Salt Lake City, UT, United States; 4 Cancer Population Sciences, Div. Oncological Science, Oregon Health & Science University, Portland, OR, United States; 5 Department of Population Medicine, Harvard University, Cambridge, MA, United States; 6 Department of Public Health Sciences, Medical University of South Carolina, Charleston, SC, United States; 7 Department of Implementation Science, School of Medicine, Wake Forest University, 525 Vine St, Winston Salem, NC, 27101, United States, 1 614-554-5298

**Keywords:** prompt engineering, few-shot learning, retrieval-augmented generation, population screening program, cancer, genetics, screening, syndrome, genomic, counseling, large language model, LLM, engineering, chatbot, prompt, RAG, mobile phone

## Abstract

The increasing demand for population-wide genomic screening and the limited availability of genetic counseling resources have created a pressing need for innovative service delivery models. Chatbots powered by large language models (LLMs) have shown potential in genomic services, particularly in pretest counseling, but their application in returning positive population-wide genomic screening results remains underexplored. Leveraging advanced LLMs like GPT-4 offers an opportunity to address this gap by delivering accurate, contextual, and user-centered communication to individuals receiving positive genetic test results. This project aimed to design, implement, and evaluate a chatbot integrated with GPT-4, tailored to support the return of positive genomic screening results in the context of South Carolina’s In Our DNA SC program. This initiative offers free genetic screening to 100,000 individuals, with over 33,000 results returned and numerous positive findings for conditions such as Lynch syndrome, hereditary breast and ovarian cancer syndrome, and familial hypercholesterolemia. A 3-step prompt engineering process using retrieval-augmented generation and few-shot techniques was used to create the chatbot. Training materials included patient frequently asked questions, genetic counseling scripts, and patient-derived queries. The chatbot underwent iterative refinement based on 13 training questions, while performance was evaluated through expert ratings on responses to 2 hypothetical patient scenarios. The 2 scenarios were intended to represent common but distinct patient profiles in terms of gender, race, ethnicity, age, and background knowledge. Domain experts rated the chatbot using a 5-point Likert scale across 8 predefined criteria: tone, clarity, program accuracy, domain accuracy, robustness, efficiency, boundaries, and usability. The chatbot achieved an average score of 3.86 (SD 0.89) across all evaluation metrics. The highest-rated criteria were tone (mean 4.25, SD 0.71) and usability (mean 4.25, SD 0.58), reflecting the chatbot’s ability to communicate effectively and provide a seamless user experience. Boundary management (mean 4.0, SD 0.76) and efficiency (mean 3.88, SD 1.08) also scored well, while clarity and robustness received ratings of 3.81 (SD 1.05) and 3.81 (SD 0.66), respectively. Domain accuracy was rated 3.63 (SD 0.96), indicating satisfactory performance in delivering genetic information, whereas program accuracy received the lowest score of 3.25 (SD 1.39), highlighting the need for improvements in delivering program-specific details. This project demonstrates the feasibility of using LLM-powered chatbots to support the return of positive genomic screening results. The chatbot effectively handled open-ended patient queries, maintained conversational boundaries, and delivered user-friendly responses. However, enhancements in program-specific accuracy are essential to maximize its utility. Future research will explore hybrid chatbot designs that combine the strengths of LLMs with rule-based components to improve scalability, accuracy, and accessibility in genomic service delivery. The findings underscore the potential of generative artificial intelligence tools to address resource limitations and improve the accessibility of genomic health care services.

## Introduction

The increased demand for genomic testing, resulting growth in patient volume, and limited access to providers with genomic expertise has necessitated new, innovative genetic service delivery models [1-6]. Prior research has demonstrated the feasibility and acceptability of incorporating technologies such as chatbots to support common communication that occurs throughout the genomic service delivery process [7-10]. Chatbots are a highly accessible and scalable platform that allows for simulated conversations. Accessible via the web through a hyperlink or downloadable app, chatbots can be used on a smartphone, tablet, or computer. The use of chatbots has also been shown to improve access to services and support health equity by providing personalized health education, being available in multiple languages, and offering continuous access to information [11-15].

The integration of chatbots into routine and ancillary tasks such as pretest counseling education, informed consent, delivery of negative results, and cascade testing have been shown to be feasible and effective in supporting genomic service delivery [8,16]. For example, chatbots have been used to collect family health history, provide pretest support, communicate with family members about results, and obtain consent for genomic research [8,17-19]. Prior results from the BRIDGE (Broadening the Reach, Impact, and Delivery of Genetic Services) trial showed equivalence between a technology-based chatbot approach and standard of care in the completion of pretest genetics education and completion of genetic testing among unaffected primary care patients meeting criteria for cancer genetic evaluation [20]. Additional research in other health service delivery contexts has found that patients using chatbots reported a better understanding of their condition or procedure, being more prepared for upcoming appointments, and feeling more informed when making health care decisions [21-29].

To date, the integration of chatbot technology into genomic service delivery has yet to focus on the return of positive genetic test results directly to patients. Currently, the return of positive results has been carried out largely through direct communication, due to the complex and sensitive nature of the information, the potential psychological impact of learning about genetic predisposition, and the need to ensure understanding of the results and their implications. However, nonchatbot technology-based solutions, such as digital patient portals, are available to communicate with patients about these results and have been shown to be highly acceptable and preferred in genomics research [8,10,16,30-35]. Furthermore, a large-scale study across 3 academic medical centers found that individuals preferred laboratory test results to be delivered immediately digitally [30].

Prior qualitative data have indicated that patients are favorable toward receiving results via chatbots, as they are convenient and allow for the opportunity to contemplate information and ask questions [8]. Digital health communication approaches, such as chatbots, may be especially appropriate for the disclosure of population-wide genomic screening (PGS) results. PGS is often conducted on a large scale, targeting asymptomatic individuals as part of public health initiatives. As a result, the communication typically emphasizes general risk awareness, with initial results disclosure indicating increased risk rather than confirming a diagnosis. The Consent and Disclosure of Recommendations workgroup funded by the National Cancer Institute’s Clinical Genome Resource (ClinGen) recommends considering factors such as test complexity, testing situation complexity, implications of genetic diagnosis to the patient and family, evidence of potential adverse psychological impact, and availability of high-quality and patient-friendly materials when deciding on the level of interaction with the patient [36,37]. Since PGS is typically completed through research and consent from participants and individuals are receiving results for well-defined hereditary conditions, the necessary level of initial communication about positive PGS results is lower than more complex, clinical results.

While high levels of acceptability, usability, and understanding of chatbots have been found in prior research, the majority of chatbots developed to date are rule-based, meaning that they operate on a set of predefined navigation paths with predefined scripted options and responses [8,9,19]. This approach allows for reliability and consistency in managing response options. However, user testing of rule-based chatbots has also revealed a need for chatbots that allow users to ask open-ended questions and receive responses in real time [8,9,19]. More recently, the release of large language models (LLMs) such as ChatGPT offers an opportunity to direct open-ended questions to LLMs to better support the return of positive genetic testing results, as open-ended questions allow for more nuanced and personalized responses. However, it is critical to test such systems to ensure that patients would receive accurate and clear information. Indeed, creating a hybrid chatbot with both rule-based and LLM components can offer a versatile and streamlined user experience by ensuring that key information is covered in the rule-based components of the chatbot and allowing for the LLM component to support complex, open-ended queries that are not covered in the scripted content. The objectives of the present project were to (1) prompt engineer an LLM-based chatbot focused on answering questions about the return of positive PGS results, and (2) conduct an intrinsic evaluation of the prompt engineering approach based on hypothetical cases and expert raters. This viewpoint paper offers insight into the application of specific prompt engineering methods to create patient-facing chatbots in the hereditary cancer diagnostic process.

## Methods

### Project Setting

We trained this chatbot using prompt engineering for the context of answering questions about the return of PGS results for an ongoing PGS program being delivered at the Medical University of South Carolina (MUSC). The PGS program was established in November 2021 with a focus on providing free genetic screening to 100,000 individuals in South Carolina. At the time of analysis, the program has recruited 59,352 individuals, returned 33,142 results, and identified 132 individuals with Lynch syndrome, 265 individuals with hereditary breast and ovarian cancer syndrome, and 191 individuals with familial hypercholesterolemia.

### Prompt Engineering Approach for Open-Ended Content

#### Overview

LLM models have been applied to improve accuracy and standardization for a variety of biomedical tasks including medical guidelines retrieval, diagnostics, medical reporting, and medical education [38-40]. The LLM selected depends on the task at hand, with a variety of LLMs developed for specific medical tasks and specialties [41]. Commonly used LLMs include ChatGPT, Perplexity AI, Claude AI, and Google Bard [42]. Developing generative artificial intelligence (AI) standards emphasizes the need to design generative AI tools responsibly for user mental models and build trust while allowing for generative variability, cocreation, and imperfection [43]. Meeting these standards requires effective prompt engineering, which includes the process of developing the text that instructs the LLM to complete a given task [44].

We used a 3-step prompt using the retrieval-augmented generation (RAG) technique which integrates retrieval-based methods with generative models, enabling the generation of contextually informed responses by retrieving relevant knowledge from a large corpus and incorporating it into the output generation process. RAG has been shown to improve LLM model performances by incorporating external information as a domain-specific knowledge base [45,46]. This project used OpenAI’s GPT Version 4-Turbo-Preview model, as new research has indicated GPT version 4 performs significantly better at answering genetics questions than version 3.5 [43,44,47]. OpenAI’s Playground was used for prompt engineering and testing. GPT4 was trained to respond about a variety of topics including providing examples of the impact of positive results, screening recommendations, and family history and cascade testing resources, and providing details regarding genetic counseling and specific PGS programs. Boundaries were also provided to ensure GPT4 responses remained within the intended scope of the chatbot.

#### Step 1: Provide Content and Context to GPT4

We used the RAG technique for prompt development. The RAG approach consisted of providing supplementary materials that were uploaded through OpenAI’s Playground “File Search” function which allows GPT4 to access the additional information in real time when responding to users’ questions. The additional files uploaded were: (1) detailed descriptions and frequently asked questions from the MUSC’s PGS website; (2) MUSC Genetic Counseling Scripts: standard scripts used by genetic counselors at MUSC, providing insights into professional communication and common queries; and (3) Genome Medical Genetic Counseling Scripts: scripts from Genome Medical to offer additional perspectives. These documents expanded the model’s knowledge base to increase the detail, consistency, and accuracy of responses. The team observed an improvement in the chatbot’s replies after including these documents based on the established evaluation criteria.

#### Step 2: Establish a Bank of Commonly Asked Questions

To train and test the LLM, a bank of commonly asked questions was developed. This bank of questions was derived from patient quality improvement interviews and expert input. This step ensures that the model is trained on a wide array of realistic and relevant scenarios, enabling it to provide accurate and helpful responses. The list of 27 questions was randomly divided into 13 training questions and 14 evaluation questions (Multimedia Appendix 1).

#### Step 3: Develop and Refine Prompts

The core of prompt engineering involves creating and refining prompts that train the AI model to elicit the most accurate and appropriate responses. The prompt development process used OpenAI GPT assistants to develop an initial draft prompt. The prompt aimed to not only inform the chatbot about the situational context and content to be discussed but also about the writing style and limitations it should adhere to. We completed iterative testing by inputting the prompt as the instructions for the AI assistant and running the 13 training questions through the messaging feature. Adjustments were made to the initial prompt until the chatbot answers were deemed accurate, clear, and appropriate by our internal team. This process is subject to the bias of the team. However, the team was careful to evaluate the chatbot responses strictly based on the evaluation criteria and quality of responses to the test questions. The prompt indicated to the LLM that patient cases would be provided as input.

### Prompt Engineering Evaluation

#### Overview

After completing the prompt engineering of our LLM chatbot, we conducted an intrinsic evaluation based on 2 hypothetical cases that were presented to domain experts in clinical genomics. The evaluation consisted of 2 steps described below.

#### Step 1: Establish the Prompt Evaluation Criteria

Previous literature has indicated relevant criteria to consider for chatbots in health communication [48]. Considering this previous work, we established relevant evaluation criteria tailored to this project through discussion and consensus among the team (Table 1). Based on 8 criteria, an evaluation instrument was developed in REDCap (Research Electronic Data Capture; Vanderbilt University) consisting of the 8 criteria, their definitions, and the ability to rate each criterion using a 5-point Likert scale from 1=Very Poor to 5=Excellent. Because prompt engineering in this context is a relatively new field, these criteria were optimized as much as possible with limited precedent.

**Table 1.** Evaluation criteria.

Criteria	Quality definition
Tone	The ability of the chatbot to express information in a way that is appropriate for the type of information being delivered
Clarity	The ability of the chatbot to communicate information clearly and in a way that avoids ambiguity or confusion
Program accuracy	The ability of the chatbot to provide correct information about the PGS^a^ program
Domain accuracy	The ability of the chatbot to provide correct information about the genetic test results and care implications
Robustness	Ability to handle ambiguous queries or incomplete information
Efficiency	Ability to provide answers that are direct, concise, and complete
Boundaries	Ability to avoid answering questions that are unrelated to the topic
Usability	Ease of interfacing with the chatbot

a PGS: population-wide genomic screening.

#### Step 2: Development of Case Scenarios and Expert Ratings

We developed 2 hypothetical scenarios focused on returning results to individuals who participated in PGS. We used scenarios to allow the expert raters to view the chatbot from the perspective of a specific hypothetical patient (Textbox 1).

Textbox 1. **Case scenarios.**

**Case 1: Anna**

- A 34-year-old White woman named Anna participated in the In Our DNA SC population-wide genomic screening program in November. She had no personal or family history of cancer. She saw a digital advertisement and decided to participate in the program out of curiosity about her health. She had previously completed a direct-to-consumer genetic screen through 23andMe but did not receive any concerning results. A few months passed after she provided her sample. Her results showed that she has a BRCA1 mutation. Anna was shocked to find this news about her BRCA1 mutation and is concerned about the next steps in her care.

**Case 2: Josh**

- A 56-year-old Black man named Josh previously had colorectal cancer. His sister participated in In Our DNA SC based on a recommendation from her doctor. Josh’s sister convinced him that it would be beneficial for him to participate. He reluctantly agreed to participate but did not see much of a point since he had already been treated for colorectal cancer. Josh received results showing that he was positive for the MLH1 gene, which means he has Lynch syndrome. Josh was confused by these results. He did not understand how these results would be important to him since he already had cancer.

## Results

### Final Prompt and Example Responses

The final prompt used for the LLM is included in Multimedia Appendix 2. Instructions for the prompt included a description of the type of patients the chatbot would be interfacing with (eg, patients who have done genetic testing as part of MUSC’s population genomics program and are receiving their results digitally), the types of questions the chatbot may receive (eg, patient questions may range from understanding their specific probability of developing cancer to inquiries about insurance coverage), and boundaries (eg, do not provide any kind of medical advice […] if the patient asks questions outside of your boundaries, reply with “I am unable to answer questions unrelated to genetic testing or helping you get to your first appointment with a genetic counselor.”). Other elements of the prompt included the style of the response and literacy level (eg, you will be replying to adults, but use a fifth-grade level vocabulary. Provide a clear, direct, concise response, preferably in bullet point format). Examples of responses provided by the chatbot are included in Table 2.

**Table 2.** Sample questions and LLM^a^ responses.

Question	LLM response
What is the cost of genetic counseling visits and the screening?	I’m here to help, but remember, I can’t give you specific costs because they can vary a lot depending on several factors. Here’s what you should know about the costs of genetic counseling visits and screenings: Insurance coverage: Many health insurance plans cover genetic counseling and screening, especially if you have a higher risk for certain conditions. However, coverage can vary, so it’s important to check with your insurance provider. Genetic counseling costs: The cost of a genetic counseling session can vary based on the location, the facility, and whether the counselor is part of a hospital or a private clinic. Some genetic counselors may work with you on payment options if you are paying out of pocket. Screening costs: Like counseling, the cost for genetic screenings can vary widely. It depends on the type of screening and whether it’s covered by insurance. Financial assistance programs: Some hospitals, clinics, and organizations offer financial assistance or programs that might help cover the cost of genetic testing and counseling for those who qualify. It’s a good idea to: Contact your insurance company to find out what they cover and if you need a referral. Ask the genetic counseling service about costs and payment options when you schedule your appointment.
What is the recommended treatment or preventative care?	For individuals with an MLH1 mutation, recommended treatment and preventive care focus on lowering the risk of cancer and catching any signs of cancer early. While I can’t give personal medical advice, here are some general recommendations: Regular screenings: Colonoscopy: Starting at a younger age and happening more often than for the general population, usually every 1 to 2 years. Physical exams: Including checking for changes in your body that might indicate cancer. Lifestyle changes: Eating a healthy diet. Staying active. Avoiding smoking. Family planning discussions: Since this mutation can be passed to your children, talking with a genetic counselor about family planning options is helpful. Consider surgery: In some cases, to prevent cancer, doctors might suggest surgery to remove certain parts of the body that are at high risk of developing cancer. This is a personal decision and requires a detailed discussion with your health care team. Stay informed: New recommendations and treatments can become available. Keep in regular contact with your health care team. A genetic counselor can provide personalized information based on your specific situation and help you understand your options for prevention and early detection. It’s important to discuss the best plan for you with your health care provider or genetic counselor. Remember, these are general recommendations, and the best approach for you should be discussed with your health care provider. If you need help scheduling an appointment with a genetic counselor at the MUSC^b^ Hollings Cancer Center Hereditary Cancer Clinic, you can visit their website or call 843-792-0745 for more personalized support.
How can I stay up to date on the latest treatments and science about my pathogenic variant?	Staying informed about the latest treatments and science related to your MLH1 mutation is a great way to actively participate in your health care. Here are some ways to keep updated: Follow reputable sources: Websites of major cancer centers, genetic organizations, and health institutions often share updates about new research and treatments. Some reliable sources include the National Cancer Institute, American Cancer Society, and the Genetic Alliance. Attend appointments: Regular check-ins with your health care team, including your genetic counselor, can keep you informed on any new recommendations or treatments relevant to your situation. Join support groups: There are support groups and communities for individuals with hereditary cancer risk. These can be great places to share information and experiences. Subscribe to newsletters: Many organizations offer newsletters that summarize the latest research and developments in cancer treatment and genetics. Educational events: Workshops, webinars, and conferences designed for patients and families can provide valuable information and updates. Ask questions: Whenever you have an appointment with your health care provider or genetic counselor, ask if there are any new findings or treatments relevant to your mutation. Remember, while it’s great to stay informed, always discuss any information or potential treatments you come across with your health care provider before making any changes to your health care plan. They can help interpret how new information may apply to your specific situation. If you’re looking for specific resources or have more questions, feel free to ask.

a LLM: large language model.

b MUSC: Medical University of South Carolina.

### Prompt Engineering Evaluation

The expert ratings of chatbot responses are provided in Table 3. Overall, the chatbot received an average score of 3.86 (SD 0.89) across all criteria and cases. The ability to express information (tone) and ease of interface (usability) received the highest scores (4.25, SD 0.68 and 4.25, SD 0.58, respectively). Boundary was rated as 4.0 (SD 0.73) followed by efficiency (3.88, SD 1.09). Clarity and robustness received scores of 3.81 (SD 1.05) and 3.81 (SD 0.66), respectively, followed by domain accuracy (3.63, SD 0.96). The lowest-rated domain was program accuracy (3.25, SD 1.39).

**Table 3.** Expert ratings for each case and combined.

Quality	Quality definition	Case 1: Anna			Case 2: Josh			Combined		
		Median (IQR)	Mean (SD)	Range	Median (IQR)	Mean (SD)	Range	Median (IQR)	Mean (SD)	Range
Tone	Ability of chatbot to express information in a way that is appropriate for the type of information being delivered	4 (4-5)	4.25 (0.71)	3‐5	4 (4-5)	4.25 (0.71)	3‐5	4 (4-5)	4.25 (0.68)	3‐5
Clarity	Ability of chatbot to communicate information clearly and in a way that avoids ambiguity or confusion	4 (3-5)	3.88 (1.1)	2‐5	4 (3-4.5)	3.75 (1.0)	2‐5	4 (3-5)	3.81 (1.05)	2‐5
Program accuracy	Ability of chatbot to provide correct information about the In Our DNA SC program	3.5 (2-4.5)	3.25 (1.58)	1‐5	3.5 (2.5-4)	3.25 (1.28)	1‐5	3.5 (2.5-4)	3.25 (1.39)	1‐5
Domain accuracy	Ability of chatbot to provide correct information about the genetic test results and care implications	4 (4-4)	3.88 (0.83)	2‐5	4 (3-4)	3.38 (1.06)	1‐4	4 (3.5-4)	3.63 (0.96)	1‐5
Robustness	Ability to handle ambiguous queries or incomplete information	4 (3-4)	3.75 (0.71)	3‐5	4 (3.5-4)	3.88 (0.64)	3‐5	4 (3-4)	3.81 (0.66)	3‐5
Efficiency	Ability to provide answers that are direct, concise, and complete	4 (3-5)	4 (1.07)	3‐5	3.5 (3-5)	3.75 (1.16)	2‐5	3.5 (3-5)	3.88 (1.09)	2‐5
Boundaries	Ability to avoid answering questions that are unrelated to the topic	4 (3.5-4.5)	4 (0.76)	3‐5	4 (3.5-4.5)	4 (0.76)	3‐5	4 (3.5-4.5)	4 (0.73)	3‐5
Usability	Ease of interfacing with the chatbot	4 (4-5)	4.38 (0.52)	4‐5	4 (4-4.5)	4.13 (0.64)	3‐5	4 (4-5)	4.25 (0.58)	3‐5
Average scores	—^a^	3.92 (3-5)	3.94 (0.92)	1‐5	3.80 (3-4)	3.88 (0.91)	1‐5	3.88 (3-5)	3.86 (0.89)	1‐5

a Not applicable.

We provided the 2 case scenarios, the test questions, and answers the chatbot had provided to those questions and were asked to rate the quality of the chatbot responses based on the designated criteria listed in Table 1. The experts independently evaluated, scored, and submitted their scores to the team. The 2 scenarios were selected to represent 2 common patient profiles that differed in age, race, gender, and background. The evaluators were aware that the responses were generated by an LLM. Eight experts completed the evaluation of the LLM output for the 2 hypothetical scenarios (Konstantinos N. Lazaridis, Libby Malphrus, Samantha Norman, Ravi Sharaf, JS, HS, Sarah English, and Anne Madeo). Experts included: 2 clinician-researchers with expertise in genomics, one genetic counselor, 3 program managers working with genomic screening programs, and 2 PhD-trained researchers with expertise in genomics. Experts were recruited based on their domain-specific knowledge and experience to provide a comprehensive evaluation of the chatbot. Descriptive statistics were calculated, including median and mean scores for each evaluation criterion.

## Discussion

### Principal Findings

We completed prompt engineering and intrinsic evaluation of the LLM component of a chatbot designed to facilitate the return of positive PGS results. Through the RAG technique, we successfully developed a prompt tailored for this application. Eight experts performed an intrinsic evaluation, which assessed the chatbot’s responses to 14 questions across 8 distinct domains in 2 hypothetical case scenarios. The chatbot achieved an overall average score of 3.88 across all domains, with the highest ratings in the tone domain and the lowest in program accuracy. These findings will inform further refinement of the prompt and integration of the LLM with the existing rule-based system, ultimately leading to the development of a hybrid chatbot to support the return of genomic screening results. As indicated by the range of scores, there was some disagreement among raters regarding the chatbot’s performance.

### Comparison to Prior Work

Prior studies have indicated that individuals are favorable toward the use of chatbots for patient follow-up and genetic test results disclosure, with a preference to include open-ended response options [8]. However, to date, few chatbots have incorporated LLMs to answer open-ended responses to questions about genetic testing in real time [8,19]. LLM responses must be carefully engineered to ensure confidence in the accuracy and reliability of responses, as well as the ability to handle ambiguous questions [49]. Our prompt engineering process resulted in a chatbot that performed well in the criteria of boundaries (ability to avoid answering questions that are unrelated to the topic), domain accuracy (ability of chatbot to provide correct information about the genetic test result and care implications), and robustness (ability to handle ambiguous queries or incomplete information). Another project focused on generative AI solutions for personalized pharmacogenomics recently identified similar trends. Prior research indicated found that the accuracy (the degree to which the responses align with guidelines) of their chatbot was rated at the 75th percentile and relevance (similar to our criteria of boundaries) was rated at the 78th percentile for patient-facing responses delivered by their chatbot [50]. These significant differences in performance metrics for these domains across responses provided by ChatGPT 3.5 and their pharmacogenomics-specific AI assistant (71st percentile vs 75th percentile for accuracy and 68th percentile vs 78th percentile for relevancy) indicate the value in prompt engineering for specific use cases. Challenges exist in ensuring domain accuracy and boundaries, such as limitations in LLM’s context retrieval and ability to process specialized biomedical and genomic data [51,52].

The combination of high domain accuracy and boundaries is essential for managing sensitive health information and mitigates concerns about chatbots offering misinformation and medical advice beyond the scope of the chatbot. As the LLM is further refined, it will be important to document all steps of the prompt engineering and be clear and transparent about the prompt engineering process used to develop the model in order to instill trust in the quality of responses and reduce the risk of misinformation [49]. It will also be critical to involve patient stakeholders in the future evaluation process. Other approaches to prompt development and evaluation include the involvement of experts (genetic counselors, oncologists) to help identify unintentional sources of bias and decide on high-quality data sources that can be used to train the model [53]. Furthermore, given that the evaluation process included only a limited set of test questions, the inclusion of a more comprehensive question set could provide additional insight into the chatbot’s performance and ensure its ability to manage a greater set of user interactions. For example, our testing included 14 questions, whereas other projects have included over 30 questions [50]. In particular, future studies should incorporate adversarial examples in both engineering and testing, especially to more comprehensively test the model accuracy and boundaries [53].

In addition to domain accuracy and boundaries, it is critical to ensure open-ended, LLM-generated responses are delivered in a tone that instills trust and engagement with the individual. Expert ratings indicated that the chatbot had good quality tone (ability to express information in a way that is appropriate for the type of information being delivered), usability (ease of interfacing with the chatbot), efficiency (ability to answer in a way that is direct, concise, and complete), and clarity (ability to communicate information clearly and in a way that avoids confusion) in both case scenarios. Prior research assessed a similar domain of language and bias (clarity and neutrality of responses, ensuring the context is understandable and devoid of bias), which was rated highly (87th percentile) [50].

### Lessons Learned

Our prompt engineering approach incorporated multiple techniques to develop an LLM chatbot that was well-rated across several quality domains. Several valuable lessons were learned. We used RAG as our approach to prompt development, but other techniques such as few-shot, supervised fine-tuning, and reinforcement learning from human feedback could be used to further adjust the model’s responses [45]. In addition, we focus on a use case of returning positive results for PGS, as PGS results return is among the least complex types of results being disclosed and could benefit from incorporating automation. Limitations of the project include our small sample size for the intrinsic evaluation of the chatbot responses and the lack of patients reviewing the responses. The reviewers are subject to bias when considering the perspective of the hypothetical scenarios which does limit the reliability of their scores.

### Future Directions

At this phase of the project, our goal was to develop the initial prompt and assess the feasibility of the prompt to respond to questions about the return of results. Thus, we did not include patients but will include patient perspectives and ratings of the quality of responses in future refinement of the LLM. Patients may identify areas for improvement that are not apparent to expert reviewers. Further, we only evaluate the script produced by the LLM component of the chatbot across 2 use cases. Additional use cases should be assessed (eg, other genes) to identify whether one prompt can be used or whether multiple prompts need to be developed for specific open-ended components of a hybrid chatbot. Finally, our assessment is only focused on the LLM component of the chatbot. Our future work will integrate the LLM component with the rule-based script, allowing us to assess different hybrid approaches. For example, we could address whether open-response options should be available as part of each component of the chatbot, which may require specific prompts for each component, or whether the open-response LLM component is generic.

While the final prompt delivered relatively high-quality responses in an appropriate tone, it is important to note that we did not assess perceptions of the quality of delivery among patients. Many chatbots have been designed to support mental health and behavior change modifications and are explicitly focused on building relationships and natural language experience for genomics-focused chatbots, and this is an important aspect of communication that will need to be evaluated before implementing a similar chatbot [54,55]. Furthermore, we tested the responses for hypothetical scenarios returning Lynch syndrome pathogenic variant (MLH1) and hereditary breast and ovarian cancer syndrome (BRCA) results. There may be a need to further refine and test response quality and tone across specific genes, as each has unique implications and may require distinct prompts. User testing among patients will also help address potential adaptations needed to ensure culturally appropriate responses [56].

Our long-term goal is to incorporate the LLM component of the chatbot described here with an existing rule-based chatbot called Genetic Risk Assessment for Cancer Education. This hybrid approach could be ideal for the return of positive PGS results, as it integrates scripted content that is critical for results disclosure with patient preference for open-ended response options. The combined approach can address the limitations of purely rule-based or purely LLM-driven systems to combine consistency and accuracy with conversational fluidity and content comprehensiveness. Some information may be more suitable for rule-based or scripted content. For example, in our intrinsic evaluation, the LLM chatbot received poor scores for program accuracy (ability of chatbot to provide correct information about the genomic screening program). The program accuracy referred to the ability of the chatbot to provide factually correct information about the specific program that patients were engaged in through this testing process. Although provided materials about the specific program were included as part of prompt engineering, experts rated this lowest among the domains they evaluated. This may indicate that additional contextual knowledge is required to sufficiently explain the complexities of individual programs. This type of information does not require personalization and may be most suited for prescripted, educational content, whereas the LLM components are most suitable for complex and open-ended questions and more nuanced interactions [49]. This additional personalization may make education more accessible and streamlined for patients seeking genetic care, potentially increasing their participation. As a result, improving the program accuracy score is an important future research topic.

One hybrid approach could incorporate a scripted component that provides a predetermined set of information, followed by an LLM component that is engineered specifically to support open-ended questions about a certain domain (Multimedia Appendix 3). This may include key domains of: overview of the PGS program, returning positive results, screening recommendations, impact on family, and next steps.

Another hybrid approach could vary when the LLM or rule-based components are used throughout the chatbot. For example, the return of results process involves 3 main stages: engagement, activation, and addressing information needs. In the engagement stage, the rule-based component of the chatbot would provide an overview of the PGS program, inform the individual of their positive results, and educate the individual about what this means for their long-term care. The activation phase could also use rule-based content and guide individuals through a core set of scripted information to encourage the next steps. In the subsequent open-ended content, participants’ information needs could be addressed by allowing them to ask additional questions about topics they choose, which could be answered through the LLM. This hybrid approach has benefits and drawbacks [57]. While the increased efficiency of resources and centralized communication are benefits of implementing the technology, the technology can introduce new types of errors, have biases of their own, and be perceived as less personable.

### Conclusions

This project demonstrated the initial feasibility of prompt engineering for the LLM component of a chatbot designed to return positive genomic screening results, with high expert ratings across most of the evaluation criteria. These preliminary findings will be used to further develop a hybrid chatbot that integrates the rule-based and LLM components to enhance the delivery of results by providing essential information with the flexibility of managing a range of patient queries. This increased efficiency has the potential to save health care systems financial and time resources. Additionally, hybrid AI tools such as these offer the potential to support patients’ decision-making and improve their education and health behaviors. Further refinements of the prompt are needed, as well as broad user-testing that involves individuals with various genomic conditions and cultural preferences, and testing of the best integration of LLM and rule-based components of the chatbot. This new approach to conveying positive genetic screening results has promise and can help address the limitations of the current genomic workforce that would be needed for the return of all positive results in a population genomic screening context.

## Supplementary material

10.2196/65848 Multimedia Appendix 1 Training and test questions.

10.2196/65848 Multimedia Appendix 2 Prompt content.

10.2196/65848 Multimedia Appendix 3 Description of PGS chat content. PGS: population-wide genomic screening.

## Abbreviations

AI: artificial intelligence
BRIDGE: Broadening the Reach, Impact, and Delivery of Genetic Services
LLM: large language model
MUSC: Medical University of South Carolina
PGS: population-wide genomic screening
RAG: retrieval-augmented generation
REDCap: Research Electronic Data Capture
