# Supplementary material for: Chatbot for the Return of Positive Genetic Screening Results for Hereditary Cancer Syndromes: Prompt Engineering Project
Source: JMIR Cancer. 2025 Jun 10;11:e65848. doi: 10.2196/65848 (PMC12172806; doi:10.2196/65848)
Supplement: Multimedia Appendix 1 [file cancer-v11-e65848-s001.docx]

| Training Questions | Evaluation Questions |
| --- | --- |
| - Are there support groups for people with similar genetic findings and how do I get in touch with them? - What exactly is a genetic counselor? - Can you explain the basic science behind how my pathogenic variant can cause cancer? - Are there additional steps that they are required to complete? (in which case I speak about the survey if it was not already mentioned) - What is my specific probability of developing cancer based on my result? - What’s the soonest I can speak to a real person? - Will insurance cover my care? - How do I schedule an appointment with a GC? - Will we be adding additional risk factors to the study? - Is this related to other cancers in my family? - What is my increased risk to develop cancer from this predisposition? - What is the best way to schedule an appointment with a genetic counselor? - What are my next steps? (i.e. surgery, screenings) | - When should I tell my insurance company about my results? - When will my results be ready in MyChart? - What is the cost of genetic counseling visits and the screening? - Is there still time for other family members to enroll and participate? - Is there anything else I need to do besides scheduling an appointment with my genetic counselor? - What is the recommended treatment/preventative care? - How can I stay up to date on the latest treatments and science about my pathogenic variant? - Who do I contact if I have questions before I am able to meet with my counselor? - What do I need to do to prepare for my first meeting with my genetic counselor? - What should I expect in my first meeting with a genetic counselor? - When/how can I view my results? - Risk to other family members? Can family members enroll in the study to have testing done as well? - Should I reach out to my primary care physician? - How can I access additional information about my variant? |
