# Supplementary material for: Chatbot for the Return of Positive Genetic Screening Results for Hereditary Cancer Syndromes: Prompt Engineering Project
Source: JMIR Cancer. 2025 Jun 10;11:e65848. doi: 10.2196/65848 (PMC12172806; doi:10.2196/65848)
Supplement: Multimedia Appendix 3 [file cancer-v11-e65848-s003.docx]

| **Component of Chatbot** | **Scripted Content** | **Open Ended Content** |
| --- | --- | --- |
| **Overview of PGS Program** | - Reminder of participation in PGS program - Introduction to PGSChat | - Details about specific PGS program |
| **Return of Positive Results** | - Disclosing results to participant - Overview of what positive results mean - Example of impact of positive results | - Additional examples of the impact of positive results |
| **Screening Recommendations** | - Screening recommendations for specific mutation | - Additional example of screening recommendations |
| **Impact on Family and Cascade Testing** | - Importance of informing family members - Summary of cascade testing options | - Additional examples of family history and cascade testing resources |
| **Next Steps** | - Description of next steps to schedule genetic counseling | - Details about what genetic counseling entails and how to prepare |
